# Puberty and NeuroDevelopment in adolescents (PANDA): a study protocol

**DOI:** 10.1186/s12887-024-05197-w

**Published:** 2024-11-26

**Authors:** Katherine O. Bray, Olivia Durbin, Stephanie Hartanto, Muskan Khetan, Daniel Liontos, Sarah J. Manuele, Isabel Zwaan, Despina Ganella, Megan M. Herting, Jee Hyun Kim, Michele O’Connell, Elena Pozzi, Orli Schwartz, Marc Seal, Julian Simmons, Nandita Vijayakumar, Sarah Whittle

**Affiliations:** 1 Department of Psychiatry, University of Melbourne, Parkville, VIC Australia; 2 Murdoch Children’s Research Institute, Parkville, VIC Australia; 3 Orygen, Parkville, VIC Australia; 4 Centre for Youth Mental Health, University of Melbourne, Parkville, VIC Australia; 5 Liggins Institute, University of Auckland, Auckland, New Zealand; 6 Department of Population and Public Health Sciences, University of Southern California, Los Angeles, CA USA; 7 School of Medicine, Institute for Innovation in Physical and Mental Health and Clinical Translation, IMPACT, Geelong, VIC Australia; 8 Department of Paediatrics, The University of Melbourne, Parkville, VIC Australia; 9 Department of Endocrinology and Diabetes, The Royal Children’s Hospital, Parkville, VIC Australia; 10 Melbourne School of Psychological Sciences, The University of Melbourne, Parkville, VIC Australia; 11 School of Psychology, Deakin University, Burwood, VIC Australia

**Keywords:** Study protocol, Adolescence, Brain, Puberty, Hormones, Emotion processing, Functional Magnetic Resonance Imaging (fMRI), Parenting, Adverse childhood experiences

## Abstract

**Background:**

Biopsychosocial changes during adolescence are thought to confer risk for emotion dysregulation, and in particular, anxiety disorders. However, there are substantial gaps in our knowledge about the biological mechanisms underlying anxiety during adolescence, and whether this contributes to the higher prevalence in females. The Puberty and NeuroDevelopment in Adolescents (PANDA) study aims to examine links between biological (sex hormones, cortisol) and social environmental factors and brain function during adolescence, with a focus on key processes (emotion regulation, fear learning) identified as relevant for the development of anxiety disorders.

**Methods:**

PANDA is a cross-sectional study with an observational design that aims to recruit a total of 175 adolescents aged 11-16 (majority female) and their parents/guardians, from the community. Brain function will be examined using magnetic resonance imaging (MRI), including functional MRI tasks of emotion regulation and fear learning. Hormones will be measured from hair (i.e., cortisol) and weekly saliva samples (i.e., oestradiol, progesterone, five across a month in females). Questionnaires and semi-structured interviews will be used to assess mental health and social environmental factors such as parenting and adverse childhood experiences. An online study of 113 adolescents was also incorporated during the COVID-19 pandemic as a questionnaire-only sub-study.

**Discussion:**

Strengths of this study include the collection of multiple saliva samples to assess variability in hormone levels, examination of the timing of adverse childhood experiences, inclusion of both maternal and paternal parental factors, exploration of mechanisms through the examination of brain structure and function, and multi-method, multi-informant collection of mental health symptoms. This study addresses important gaps in the literature and will enhance knowledge of the biological and environmental contributors to emotion dysregulation and anxiety in adolescents.

**Supplementary Information:**

The online version contains supplementary material available at 10.1186/s12887-024-05197-w.

## Introduction

Anxiety disorders are the most common of all mental health disorders worldwide [1, 2]. Onset is early in life, with 52% of cases diagnosed by the end of adolescence [3]. Females are almost twice as likely to suffer from an anxiety disorder as compared to males, with sex differences appearing during adolescence [4]. Critically, childhood and adolescent onset of anxiety disorders lead to more severe impairment across the lifespan, compared to adult onset [5]. Despite a clear clinical need, adequate treatments for anxiety disorders in youth are lacking, with over 50% of young people with anxiety disorders not responding to first-line treatments (e.g., cognitive behavioural therapy involving exposure training; [6].

Dramatic hormonal changes and brain development occur during adolescence, and these changes render individuals at risk for impaired emotion processing, and in particular, vulnerability to anxiety disorders [7]. However, there are substantial gaps in our knowledge about the biological mechanisms underlying anxiety disorders during adolescence, and whether this contributes to the higher prevalence in females. It has been proposed that knowledge of the biological mechanisms of anxiety is crucial for progress in identifying effective treatments [8].

### Neurobiological mechanisms of anxiety

During adolescence, there is marked development of the neural circuitry underlying emotion processing and regulation, including the amygdala and prefrontal cortex (PFC). Around the start of puberty, there are changes to bottom-up emotional processing in the amygdala and top-down regulation from prefrontal regions. This imbalance between emotional processing and control systems may play a role in the increased risk for anxiety and other affective disorders during this period [9].

#### Emotion regulation

Emotion regulation refers to goal-directed cognitive and behavioural processes that influence the intensity, duration and type of emotional experience and expression [10]. Emotion regulation difficulties during adolescence are widely held to be a transdiagnostic risk factor for internalising disorders [11]. Connections between the amygdala, hippocampus and PFC comprise the core circuitry involved in emotion regulation [12]. In adolescents, compared to children and adults, higher subcortically-driven emotion reactivity coupled with under-developed capacity to regulate emotional responses via prefrontal mechanisms result in relatively immature emotion regulation [13, 14]. While individual differences in the development of brain regions subserving emotion regulation predict risk for a range of mental health difficulties, including anxiety [15–19], further research is needed to directly investigate alterations in the neural correlates of emotion regulation that are related to anxiety in adolescents.

#### Fear learning

There is consensus that impaired fear learning processes, and specifically, fear extinction recall (FER), are characteristic of anxiety disorders [20, 21]. FER refers to the retrieval and expression of a learned extinction memory (i.e., a learned memory that a previously feared stimulus is no longer a threat). The neural correlates of FER are well characterised in adults [22]. However, as compared to emotion regulation, limited research has been conducted to investigate the neural mechanisms of FER in adolescents. Adolescent rodents show deficits in FER, which may underlie a general vulnerability to anxiety during this developmental period [23, 24]. Dysfunction of the ventromedial PFC (vmPFC) is a key mechanism underlying deficits in adolescent FER in rodents. Our pilot work provides evidence for differences in the neural underpinnings of FER in adolescents versus adults, particularly involving the vmPFC and its connectivity to subcortical brain regions [25, 26]. In addition, sex differences were found, with female adolescents showing reduced vmPFC activity compared to males during extinction. However, given the small sample in our pilot work (*n* = 17 adolescents, 10 female), further work is needed to understand the association between adolescent sex differences in susceptibility to anxiety and neural correlates of FER.

### Puberty and the impact of hormones on neurodevelopment

Two decades of research has clearly demonstrated that brain development is driven in part by pubertal changes [27–31]. However, most research has focused on adrenal hormones or testosterone. Relatively little research has investigated the role of other sex hormones, including oestradiol (E2) and progesterone (P4). Although changes in these hormones occur in both males and females, they are a defining feature of female puberty. The onset of gonadarche (also known as ‘puberty proper’, driven by activation of the hypothalamic pituitary adrenal axis) occurs at approximately 10 years of age in females (though there is wide variation; [32] and begins with renewed secretion of gonadotropin-releasing hormone from the hypothalamus. Gonadotropin secretion (i.e., luteinising hormone and follicle stimulating hormone) from the pituitary gland in turn stimulates the release of gonadal sex hormones E2 and P4 from the ovaries, and the development of secondary sex characteristics. E2 and P4 are considered neurosteroids as they are centrally converted and synthesised de novo by the brain [33]. These neurosteroids play a role in regulating neurotransmitter systems [34]. Regions of brain networks involved in emotional regulation and FER (e.g., amygdala, PFC) are also densely innervated by neurosteroid receptors [35]. Neurosteroids act in the central nervous system by modulating the synthesis, release, and metabolism of different neurotransmitters and neuropeptides to influence the excitability, synaptic function, and morphological characteristics, i.e., growth of neurons [36, 37]. It is through these biochemical changes that E2 and P4 are suggested to have both activational and organisational effects on the brain during adolescence [38, 39].

While these hormones play a role in male physiology, their cycling nature and contribution to female puberty and menstruation is unique. In regularly cycling adult females, phases of the cycle where E2 levels are high are associated with superior emotion regulation states [40]. During transitional periods (i.e., puberty, menopause), where cycles are irregular and difficult to track, the degree of short-term hormonal variability is suggested to be critical for emotional functioning [41]. In particular, greater within-month, week-to-week variability in E2 has been associated with increased negative mood, including in pre-menarcheal adolescents [42]. Adding another layer of complexity, it has been shown that sex hormones can have opposing effects during transitional vs. non-transitional periods. For example, while in reproductive-aged adults E2 exerts both anxiolytic and antidepressant effects on the brain via its GABAergic effects [41], rodent research suggests that during puberty, E2 appears to be anxiogenic and disrupts FER, perhaps due to irregular oestrous cycling [43, 44]. Accordingly, it may be that increased hormonal fluctuation (i.e., variability) acts on the brain and impacts emotional processing in ways that worsen mental health.

#### Effects of E2 and P4 on the adolescent brain – evidence from human Magnetic Resonance Imaging (MRI)

Although there are some inconsistencies, a handful of cross-sectional studies have provided evidence that E2 and P4 are associated with brain structure and function in female adolescents. As discussed in recent reviews [31, 45], studies have found E2 levels to be associated with reduced grey matter in adolescent females, particularly in PFC regions. Functional MRI (fMRI) studies have provided some evidence that E2 modulates subcortical and prefrontal activity during emotion processes, such as cognitive reappraisal of emotional stimuli [46]. We have recently found that increased week-to-week variability in E2 levels in adolescent females (both pre- and post-menarche) was associated with reduced thickness in the vmPFC [47]. Beyond this limited investigation into the effects of mean and fluctuating hormone levels, no published studies to date have investigated the effects of short-term variation in female sex hormones on emotional brain function.

#### HPA axis function

The dramatic alterations in pubertal hormones seen with the onset of puberty (triggered by activation of the hypothalamic–pituitary–gonadal axis) is thought to have a modulatory role in hypothalamic–pituitary–adrenal (HPA) axis function, potentially contributing to vulnerability for anxiety and depression in adolescents [48, 49]. The HPA axis plays a central role in the response to stress and produces cortisol (the human stress hormone), which can easily pass through the blood-brain barrier and readily interacts with the amygdala, PFC, and their connections [50]. It is via this mechanism that cortisol is thought to be important for emotion regulation and fear learning. Cortisol has been found to modulate emotion regulation neural circuitry [51]. It has also been shown to influence brain activation during fear learning in a manner that differs by sex [52, 53]. However, much of this work has been performed in adults and further research is needed to understand the role of the HPA axis in influencing emotional brain function in adolescents.

### Social environmental factors as context

Additional factors, such as the social environment, also impact the brain and subsequently, mental health. Indeed, social environmental factors (e.g., early adverse experiences) are some of the strongest predictors of mental illness [54]. It is theorised that such factors may influence mental health via influencing brain development [55–57].

#### Adverse childhood experiences

Adverse childhood experiences (ACEs) encapsulate a range of distressing and potentially traumatic childhood events, including exposure to violence at home and in the community, experiences of bullying, and household dysfunction (e.g., witnessing ongoing parental conflict; [56–63]. Such experiences have been documented to substantially increase the risk for mental health conditions, including anxiety, with effects that may persist beyond the early developmental years and into adulthood [64–68]. The mechanisms underlying how ACEs contribute to poor outcomes are yet to be fully understood; however, researchers suggest that ACEs may induce structural and functional brain alterations, particularly in regions and circuitries that are implicated in stress and emotion regulation processes [68–70]. Theoretically, it has been suggested that these alterations are induced through enduring activation of the HPA-axis and the subsequent release of cortisol upon perception of stressors [71, 72]. Indeed, a body of literature has highlighted the associations between ACEs exposure and HPA-axis dysregulation [72]. The effects of ACEs on the brain, however, may vary depending on the developmental timing of exposure. Studies suggest that exposure at different ages or developmental stages (e.g., childhood versus adolescence) is associated with differential structural and functional brain outcomes [71]. Empirical work testing timing effects on the brain, however, is scarce, and it is unclear whether timing of ACEs impacts the neural circuitry underlying emotion processing, with implications for anxiety and other mental health problems.

#### Parental factors

Parenting behaviours are thought to impact the way in which children develop both neurobiologically and psychologically. Negative parenting behaviours (e.g., rejection, psychological control, harsh discipline) have been consistently associated with increased risk for mental health problems, including anxiety [73, 74]. Conversely, low levels of positive parenting behaviours (e.g., warmth, acceptance) have been associated with similar poor child and adolescent outcomes [73, 74]. Additionally, studies have suggested that exposure to parental anxiety and/or depressive problems is associated with increased risk of child and adolescent development of internalising problems [75]. A growing body of evidence points to alterations in brain development as one mechanism by which parental factors may influence child and adolescent mental health. Functional neuroimaging studies, for example, have found parenting behaviours and parental mental health problems to be associated with brain function and connectivity during emotion processing and regulation in corticolimbic regions/circuits (e.g., [76]). One significant limitation of this body of work, however, is the lack of systematic investigation into the potentially differential roles of various caregivers. Mothers are over-represented in the parenting literature in general. Our recent review suggests that paternal caregiving is as important as maternal caregiving behaviours in predicting adolescent anxiety and depression, and in some cases maternal and paternal parenting may interact to influence mental health [77]. Similarly, studies have demonstrated associations between both maternal and paternal anxiety and depressive problems and increased risk of adolescent development of mental health problems, although these findings remain relatively inconsistent [78, 79]. Research is needed to understand the potentially unique and interactive contribution of maternal and paternal factors in influencing adolescent neural circuitry underlying emotion processing.

### Rationale and aims

There are substantial gaps in our knowledge about the biological mechanisms underlying anxiety disorders in adolescence, and how they might contribute to the higher prevalence in females. Knowledge of the biological mechanisms of anxiety is crucial for progress in identifying effective treatments [8]. There are methodological limitations in the existing literature on female sex hormones and brain development. Despite the importance of female sex hormones for emotional behaviour, there is a lack of human neuroimaging research investigating their impact on the developing brain. We contend that understanding how key characteristics of anxiety are represented in the adolescent brain, and how these representations are moderated by sex, hormone levels and social environmental factors, is a fundamental imperative to improve health and functional outcomes for young people.

**Fig. 1:**
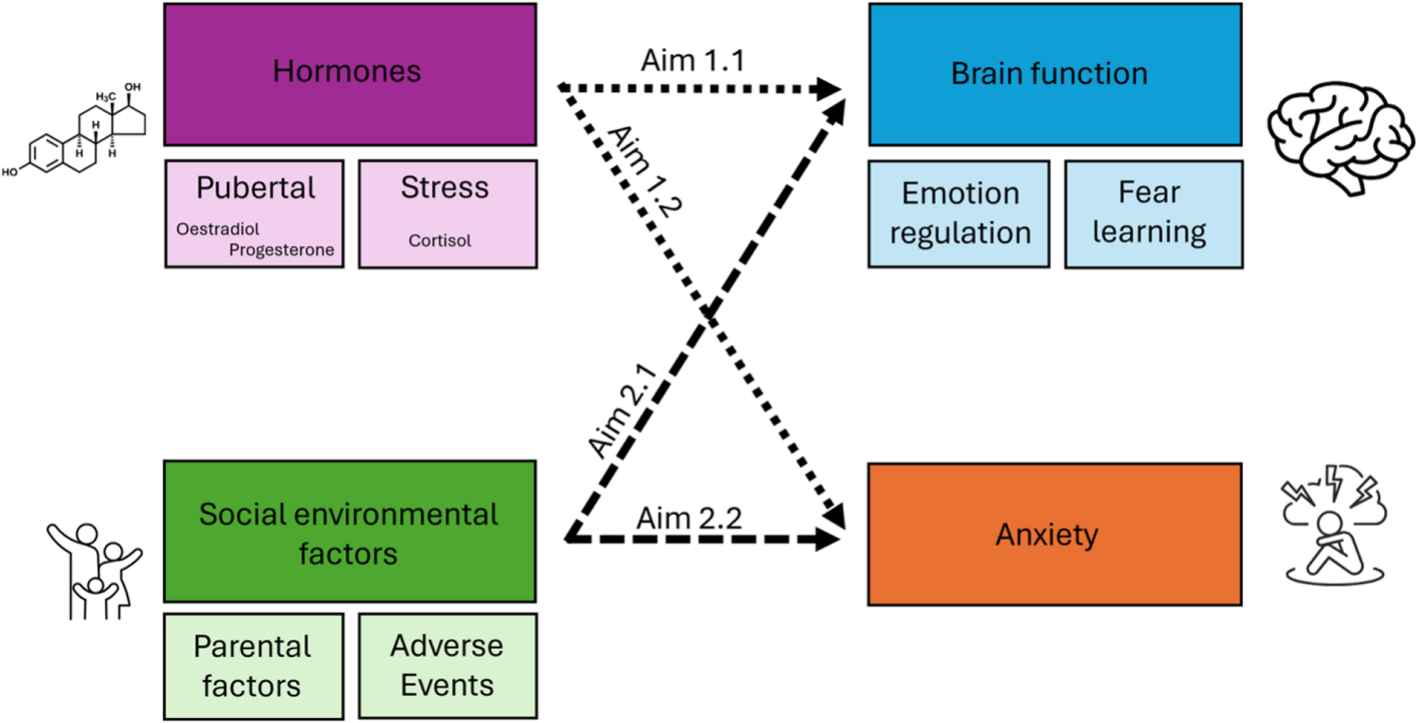
Conceptual model of the key elements of the Puberty and NeuroDevelopment in Adolescents (PANDA) study

This project aims to investigate the role of both biological (sex hormones, cortisol) and social environmental (adverse child experiences, parental) factors in the neural mechanisms underlying several key processes (emotion regulation, fear learning) identified as relevant for the development of anxiety disorders, and concurrent anxiety symptoms (see Fig. 1).

#### Specific aims

1. *To examine the effects of sex hormones (i.e., E2 and P4) on brain function and anxiety during adolescence*

It is hypothesised that greater short-term (week-to-week) variability in E2 and P4 in females will be associated with reduced FER and altered vmPFC activity and connectivity. Greater variability will also be associated with altered amygdala and PFC activity and connectivity during emotion regulation, and with higher anxiety symptoms.

Exploratory analyses will also investigate how (a) mean E2 and P4 levels relate to brain function and anxiety symptoms, (b) the association between hormones and brain function is moderated by age, (c) hormones relate to emotional states as assessed via repeated sampling of affect, (d) hormones relate to brain structure, and (e) FER and emotion regulation brain function differs between males and females.

2) *To examine how social environmental factors (parental factors [parenting behaviour and parent psychopathology] and ACEs) impact brain function and anxiety during adolescence.*

It is hypothesised that paternal and maternal parenting behaviour and psychopathology will be associated with PFC and amygdala activity and connectivity during FER and emotion regulation in adolescents, and anxiety symptoms. We hypothesise that paternal and maternal factors will have both independent and interactive effects.

We hypothesise that ACEs will be associated with PFC and amygdala activity and connectivity during FER and emotion regulation, and anxiety symptoms, with these associations moderated by timing of ACE exposure.

Exploratory analyses will also investigate how (a) pubertal hormones and social environmental factors interact to predict brain function and anxiety symptoms, (b) social environmental factors relate to brain structure, and (c) social environmental effects on brain function and anxiety differ between males and females.

## Methods

### Overall study design

The Puberty and NeuroDevelopment in Adolescents (PANDA) study is a cross-sectional, observational, community-sample study based at the University of Melbourne, Australia, with MRI scans and onsite procedures occurring at the Royal Children’s Hospital, Australia. Recruitment and assessment procedures for the main arm of the study began in June 2022 and are anticipated to be completed mid-late 2024. Due to disruptions and delays caused by the COVID-19 pandemic, an additional online-only arm of the study was conducted between September 2021 and March 2023. This arm of the study was added to increase power to address Aim 2.2 (Fig. 1). Ethics approval was granted through the Royal Children’s Hospital Human Ethics Research Office (#1955580). The study adhered to the ‘strengthening the reporting of observational studies in epidemiology’ (STROBE; www.strobe-statement.org) guidelines. See Supplementary Materials for STROBE cross-sectional study checklist. Funding was obtained from the National Health and Medical Research Council (GNT1163499) and Australian Research Council (DP220102135).

### Participants

Participants of the main “in-person” study will be 175 adolescents, aged 11 to 16, and their parents/guardians. As a key aim of our in-person study is to understand the impact of the sex hormones E2 and P4 and their fluctuating levels across time in females, we will oversample female adolescents (*n* = 145). This will serve to increase power to examine the effects of hormone variation in females. A sample of 30 male adolescents will be recruited for comparison purposes. Additionally, adolescents living anywhere in Australia aged 12 to 16, and both their male and female parents/caregivers were recruited for the online questionnaire-only study (completed). A lower limit of 12 years was adopted given concerns about obtaining informed assent in minors without face-to-face contact. 113 families (54% female adolescents) participated in the online study, with an additional 42 families providing at least one family member response (maternal caregiver: 26; paternal caregiver: 7; adolescent males: 2; adolescent females: 7). Please see Table 1 for full eligibility criteria for both study streams.

**Table 1:** Eligibility criteria

Inclusion Criteria	Exclusion Criteria
Adequate comprehension of written and spoken English;	Diagnosis of a developmental disorder (e.g., attention-deficit/hyperactivity disorder, autism spectrum disorder, or intellectual disability);
Living in Greater Melbourne, or able to travel to the Royal Children’s Hospital ^a^;	Medical or neurological condition for which adolescent is taking psychoactive medication ^a^;
Aged between 11–16 years old at the time of participation (12–16 for online only study);	Current or previous pregnancy;
Assent provided by adolescent for their participation and consent provided by a parent/caregiver for their own and their child’s participation;	Loss of consciousness for 5 min or more as the result of head injury ^a^;
Involvement from both a male and female parent/caregiver that have been in the adolescent’s life for > 3 years ^b^	Any contraindications to Magnetic Resonance Imaging: for example, metal on/in their body that cannot be removed (e.g., piercings, braces, implantable devices)^a^;
	For post-menarcheal females, hormone-based contraceptive use ^a^;
	Diagnosis of an endocrine disorder, premature adrenarche or precocious puberty, or reported use of hormonal medications (for example chronic use of corticosteroids [e.g. Hydrocortisone], or gonadotropin-releasing hormone agonists [e.g., Lypron]) ^a^;
	Body mass index < 5th or > 95th percentiles ^a^; Adolescents whose siblings have previously participated in the study.

Inclusion and exclusion criteria denoted with an ^a^ did not pertain to the online sub-study

Inclusion and exclusion criteria denoted with an ^b^ solely pertain to the online sub-study

### Data collection procedures

#### Recruitment

##### In person study

An array of recruitment methods is being employed including:

- Social media platforms, with advertisements targeting adolescents or parents of adolescents (i.e., Facebook, Instagram, see Supplementary Materials for more information).
- BrainBee (a science competition where the regional finals take place in Melbourne).
- Independent (i.e., non-government) schools in metropolitan Melbourne.
- Snowball sampling utilising word of mouth and participant referral.

Advertising materials (e.g., posters, newsletter inserts, social media posts) contain a link to the study website which comprises: more information about the study; a list of inclusion and exclusion criteria and contact information for the study team. Parents of interested families receive a copy of the participant information and consent form and arrange a phone call with researchers, who obtain verbal consent for both the adolescent and their parent and complete an eligibility screening questionnaire (see Table 1 for eligibility criteria).

##### Online study

For the online study, recruitment methods also included utilising social media, snowball sampling and independent schools as described above. Families could be living anywhere within Australia. Advertising materials contained a link to a screening questionnaire (determining child age, presence of a second caregiver, and informed consent). The primary caregiver, who completed the screening questionnaire, was asked to provide email and phone details of their child and the child’s second parent/caregiver. The questionnaire was subsequently emailed directly to each family member.

#### Data collection overview

##### In person study

Participant data is collected through a combination of at-home tasks and an in-person visit to the Royal Children’s Hospital, Melbourne, Australia. Participant information and questionnaire response data is collected using REDCap and Qualtrics software. Assessment begins with at-home tasks including the collection of weekly saliva samples at wake, leading up to the date of the adolescent’s in-person visit (see Measures for more information). Approximately one week prior to the in-person visit, researchers also conduct an online clinical interview with adolescents, where a mental health assessment is administered by trained research personnel under the supervision of a clinical psychologist. Adolescents are then asked to complete a series of questionnaires at home (see a detailed summary of questionnaires listed in Table 2) prior to their in-person appointment.

Adolescents and their caregivers are asked to bring their frozen saliva samples to their in-person visit at the Royal Children’s Hospital. At this visit, adolescents and their parents begin by providing written assent/consent and are subsequently taken through a mock scan in preparation for the MRI. During the mock scan, adolescents lay supine in the practice MRI scanner and are familiarised with the procedure, including receiving instructions for the fMRI tasks. Adolescents then participate in an MRI brain scan where they complete a structural scan, a resting state functional sequence, and either one or two fMRI tasks depending on their age group (see Measures for more information). Researchers also collect anthropometric measurements (height, weight, and waist circumference) and a hair sample. At the end of the in-person visit, researchers conduct a debriefing interview with participants where adolescents are provided with a summary of their activities and mental health resources. Participants are reimbursed for their time and provided with a personalised 3D model of their brain.

##### Online study

The COVID-19 pandemic presented accessibility and recruitment constraints which suspended the in-person study. Due to researchers’ lack of access to hospital-based MRI scanners, an online study was conceptualised to capture questionnaire data from adolescents and their primary male and female caregivers, Australia-wide. This online-only sub study was performed to boost power for addressing Aim 2.2 (see Fig. 1). The screening questionnaire commenced with an information statement and required electronic signature to proceed. To ensure that adolescent participants understood the purpose of the study, and the nature of the questionnaires, they had to correctly answer three questions assessing their comprehension before written consent was obtained (see Supplementary Materials for questions).

**Table 2:** Summary of measures collected for In-Person and online cohorts, reported by adolescents, and their Parents/Caregivers

Measure	CSR	PSR	CPR	PCR
***Anthropometry***				
Edinburgh Handedness Inventory - Short form	✓			
Height ^a^	✓			
Waist circumference ^a^	✓			
Weight ^a^	✓			
***Mental Health Interview***^***a***^				
Mini-International Neuropsychiatric Interview for Children and Adolescents	✓			
***Hormonal Sampling***^***a***^				
Hair sample	✓			
Saliva samples	✓			
***MRI Brain Scan***^***a***^				
Affecting labelling fMRI task	✓			
Fear learning fMRI task ^b^	✓			
Resting fMRI task	✓			
Structural MRI scan	✓			
***Anxiety and Mood-Related Questionnaires***				
Beck Anxiety Inventory		✓*		
Brief Multidimensional Students’ Life Satisfaction Scale	✓			
Center for Epidemiologic Studies Depression Scale		✓*		
Child Depression Inventory − 2	✓*			✓*
Childhood Anxiety Sensitivity Index	✓			
Difficulties in Emotion Regulation Scale	✓			
Positive and Negative Affect Schedule	✓			
Spence Children’s Anxiety Scale- Short version	✓*			✓*
State-Trait Anxiety Inventory		✓*		
State-Trait Anxiety Inventory - Children	✓*			✓*
Strengths and Difficulties Questionnaire - Externalising				✓*
Visual Analog Scale - Assessment of anxiety surrounding MRI scan	✓			
***Puberty Questionnaires***				
Pubertal Development Scale	✓*			
***Attachment and Parenting Questionnaires***				
Children’s Perceptions of Interparental Conflict			✓*	
Egna Minnen Betraffande Uppfostran [My Memories of Upbringing]		✓*	✓*	
Experience in Close Relationships - Short Version (Closeness in Romantic Relationships)		✓*		
Experience in Close Relationships for Children - Short Version (Closeness to Parents)	✓*			
Inclusion of Other in Self - (Child to Parent)	✓*			
Inclusion of Other in Self - (Parent to Spouse)		✓*		
Parental Acceptance-Rejection Questionnaire		✓*	✓*	
***Adversity Questionnaires***				
Maltreatment and Abuse Chronology of Exposure Parent Report and Child Report	✓			✓
***Social-emotional Questionnaires***				
Adolescent Measure of Empathy and Sympathy	✓			
Empathic Distress Questionnaire	✓			

*CSR *Child self-report, *PSR *Parent self-report, *CPR *Child report on parent, *PCR *Parent report on child

*Presence of the measure in the online study

^a^Collected by, or with the assistance of researchers. Note that for height, weight and waist circumference and saliva, parents may assist

^b^Tasks completed by 14- to 16-year-old participants only

### Measures

#### Demographics

We collect information on adolescent sex and gender, household income, parental occupation and family structure. Please see Supplementary Materials Table S1 for the full list of all demographic questions asked.

#### Mental health interview

Adolescents participate in an online mental health interview (~ 30 min) approximately one week prior to their in-person visit. The interview comprises the administration of the Mini-International Neuropsychiatric Interview for Children and Adolescents (MINI-KID, version 7.0.2, consistent with the DSM-V [80] with a researcher, and the completion of the Maltreatment and Abuse Chronology of Exposure [81]. Note that we administer all modules of the MINI-KID, except for Module B: Suicidality and Suicidal Behaviour Disorder. Instead, Module A: Major Depressive Episode and Major Depressive Disorder is used as a screener for a comprehensive assessment of risk for suicidality by the researcher. Additionally, Module X: Autism Spectrum Disorder is not administered as it has been pre-emptively screened as exclusion criterion, and the module’s outcome does not provide conclusive grounds for exclusion.

#### Questionnaire measures

See Table 2 for a summary of the questionnaire measures collected and Supplementary Materials for in depth-description of the questionnaire measures. Questionnaire measures administered to adolescent participants include handedness, anxiety and depressive symptoms, mood, pubertal development, child-parent attachment and closeness, perceived parenting behaviours and interparental conflict, experiences of adversity and social-emotional functioning. Questionnaire measures administered to the parents/caregivers include child externalising behaviours, their own depressive and anxiety symptoms, and romantic attachment and closeness. Note that adolescents nominate their primary and secondary caregiver. The order in which parenting behaviour questionnaires are presented to the adolescents is randomised across the sample, to ensure counterbalanced presentation of questions pertaining to the nominated primary caregiver and the nominated secondary caregiver, to reduce response bias. Adolescents in single parent households are not presented with questionnaires regarding a secondary parent/caregiver or interparental conflict.

#### MRI brain scan

##### MRI brain scan parameters

Neuroimaging data is acquired on the 3T Siemens TIM Trio scanner (Siemens, Erlangen, Germany) at the Royal Children’s Hospital. Participants lie supine with their head supported in a 32-channel head coil.

*Structural Scan* – Adolescent participants undergo a T1-weighted structural scan in the MRI scanner whilst they watch part of a film. A software upgrade occurred after the first 23 participants. This resulted in slightly different sequence parameters for the T1-weighted scan before versus after the upgrade (details below). Prior to the upgrade: T1-weighted 3D MPRAGE images were acquired with motion correction with the following parameters: repetition time = 2550ms; echo time1 = 2.14ms; echo time2 = 3.94ms; echo time3 = 5.77ms; echo time 4 = 7.5ms; flip angle = 8°; field of view = 256 mm; ) producing 208 contiguous 0.9 mm thick slices (voxel dimensions = 0.9mm^3^). Sequence duration 6:47 min. Since the upgrade: T1-weighted 3D MPRAGE images are acquired with the following parameters: repetition time = 2500ms; echo time1 = 1.72ms; echo time2 = 3.45ms; echo time3 = 5.18ms; echo time 4 = 6.91ms; flip angle = 8°; field of view = 256 mm; producing 208 contiguous 0.9 mm thick slices (voxel dimensions = 0.9mm^3^). Sequence duration = 7:32 min.

*Resting state fMRI* – Adolescents participate in a resting state functional scan where they are required to fixate on a cross symbol and let their mind wander while staying awake. Exact instructions: “keep your eyes open, look at the cross, try not to think about anything in particular, you can let your mind wander. Try not to fall asleep”. Resting state fMRI data are collected with whole-brain 2D echo planar imaging with a multiband acceleration factor of 3, TR = 1300 ms, TE = 33 ms, flip angle = 85°, field of view = 255 mm^2^, to produce 60 2.5 mm thick interleaved slices (voxel dimensions 2.5 mm^3^). Sequence duration = 6:06 min.

*FMRI tasks* – fMRI task data are collected with T2*-weighted echo planar imaging images with the following parameters: multiband acceleration factor of 3, TR = 1250ms, TE = 30ms, flip angle = 90◦, field of view = 2552 mm; producing 60 2.5 mm slices (voxel dimensions 2.5 mm3). The total sequence is 8:12 min for the affect labelling task and 15:34 and 9.41 for part one and two of the fear learning task, respectively. When participants are required to make a response during the tasks they use a 4-button Fiber Optic Response Pad (Current Designs). Auditory stimuli, including task instructions, are delivered through MRI-compatible headphones.

**Fig. 2:**
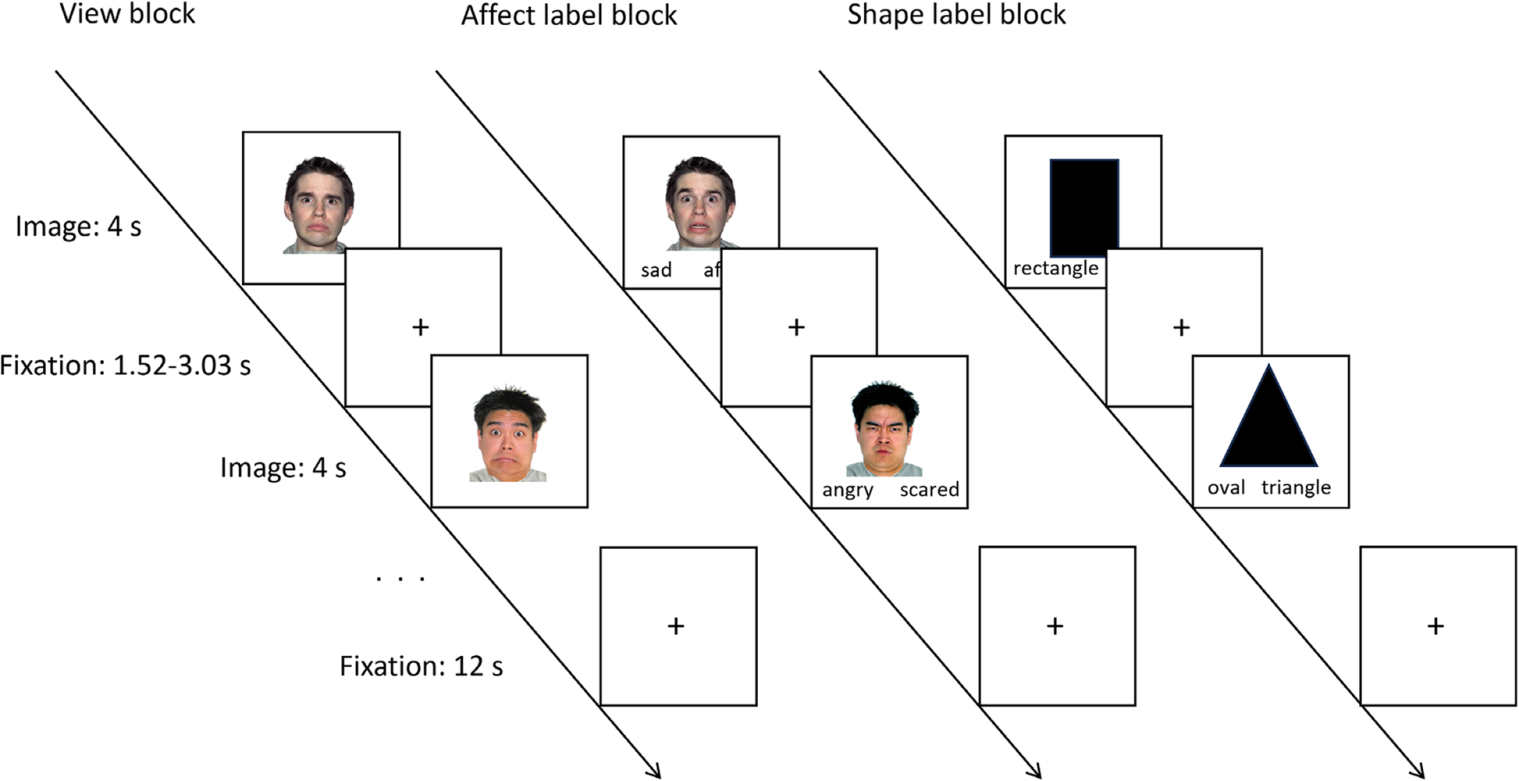
Affect labelling functional Magnetic Resonance Imaging task design. Affect labelling functional Magnetic Resonance Imaging task design. Sequence and timing of stimuli presented in view, affect label and shape label blocks. Note: s = seconds

*Affect labelling fMRI task* - Adolescent participants are administered an adapted implicit emotion regulation task [82], involving the presentation of negative (sad, fear, angry) facial stimuli (from the NimStim set [83], which requires them to either label or view the expressions (see Fig. 2). The task also includes a shape labelling condition where adolescents match shapes presented with one of two labels, to control for the potential confound of cognitive processing involved in the matching task. Participants complete a short practice version of the task with a researcher prior to their MRI scan, where they are able to trial the labelling and viewing conditions. During a ‘label’ condition, participants are instructed to match the facial expression or shape shown with one of two labels, found at the bottom left and right of the screen respectively. During a ‘view’ condition, participants are instructed to view the face and press their thumb to the side of the button box to control for the potential confound of motor activity. The label and view conditions are presented randomly in a total of 10 blocks, with two blocks of the shape label condition, and 4 blocks of the face label and view conditions. Each block comprises six 4 s (s) trials, interspersed by a jittered fixation of 1.52–3.03 s. A fixation cross is presented for 12 s between each block. Faces are all male and are balanced for race (two-thirds White and one-third Asian to reflect ethnic representation in Australia). Emotional labels include afraid, angry, sad, miserable, mad, and scared; and shape labels include triangle, rectangle, and oval.

**Fig. 3:**
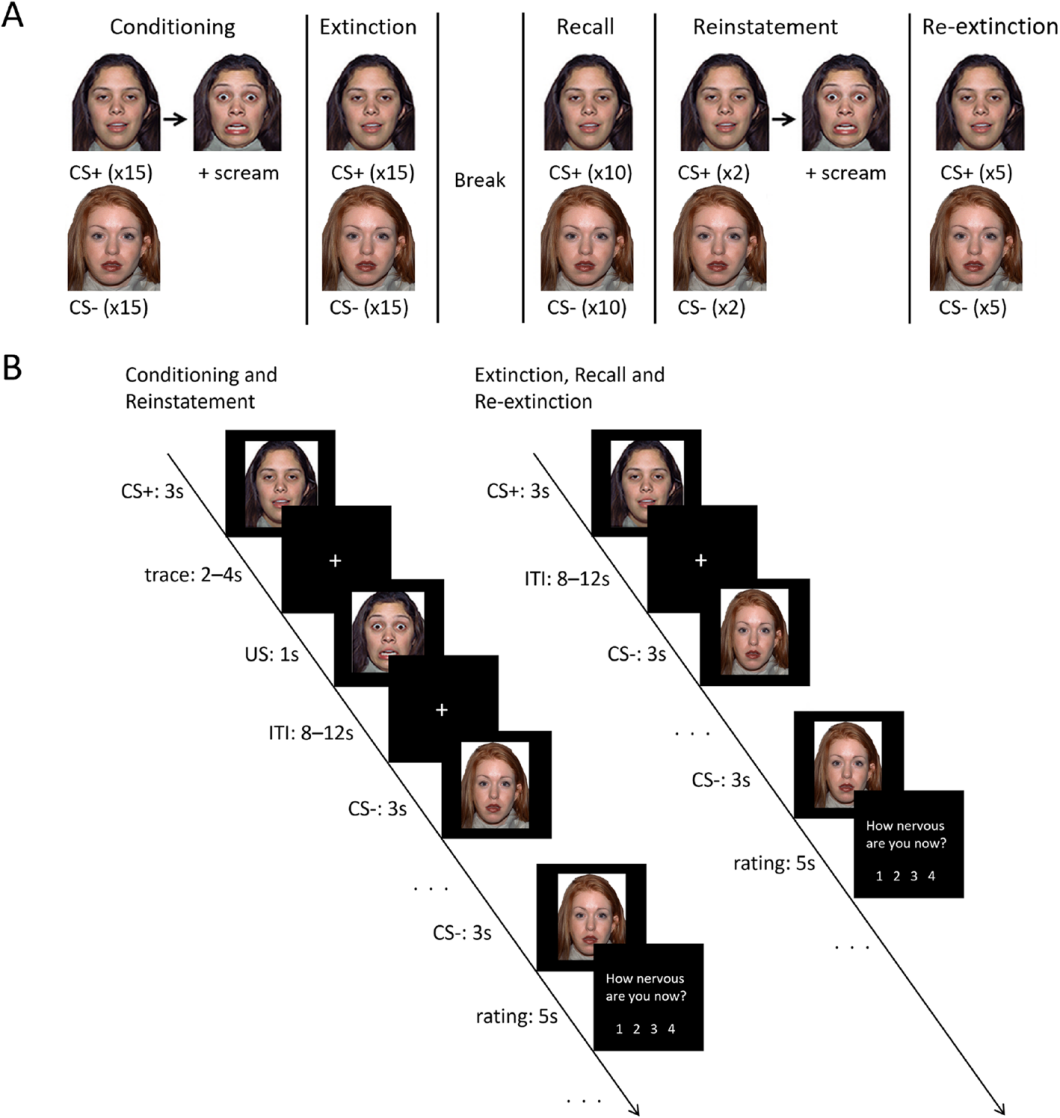
Fear learning functional Magnetic Resonance Imaging task design. **A **Overall schematic of the task phases. Part one of the task includes fear conditioning and extinction learning. Part two of the task includes extinction recall, reinstatement and re-extinction. x2, x5, x10 and x15 refer to the number of stimuli presented during each task phase. **B** Sequence and timing of stimuli presented in different phases. Note: CS+ = conditioned stimulus paired with the US, CS- = conditioned stimulus never paired with the US, ITI = inter-trial-interval, s = seconds, US = unconditioned stimulus

*Fear learning fMRI* task - Adolescent participants aged 14–16 are administered a fear learning task (see Fig. 3) divided into two parts; the first immediately after the structural scan, and the second as the final task of the MRI scan. Younger participants aged 11–13 do not participate in this fMRI task due to potential distress from the scream sounds in this younger age group (see Supplementary Materials for further information).

During the first part of the task, participants undergo pre-conditioning/familiarisation, fear conditioning and extinction learning phases. All faces presented in the task are female faces from the NimStim set. During pre-conditioning (not shown in Figure), participants are presented with two female adult neutral faces (counterbalanced across participants) and played a scream sound through headphones. During conditioning, one of the two neutral faces (conditioned stimulus positive; CS+) is presented for 3 s followed by 2–4 s jittered trace period then a fear face (1 s) and female scream (unconditioned stimulus; US, ~ 95–100 dB). The CS + is reinforced 100% with the US during conditioning based on other human fear trace conditioning studies, see [22, 84] for review. The other face (conditioned stimulus negative; CS−, 3 s) is used as a control stimulus that is never paired with the US. CS + US trials and CS − trials are interleaved and are presented 15 times each. The inter-trial interval is a white fixation cross on a black background, jittered for 8–12 s. Following conditioning, participants are presented with each CS and asked to rate how nervous they feel (1 = least nervous to 4 = most nervous). CS’s are presented for 3 s and participants have 5 s to respond. Extinction follows immediately, with presentation of 15 CS+ (3 s) trials in the absence of the US, interleaved with 15 CS− (3 s) trials. Extinction concludes with the same ratings of nervousness.

After extinction, there is a ~ 15-minute rest prior to the second part of the task (extinction recall, reinstatement, re-extinction), based on similar timing used in previous studies [26, 85, 86]. During extinction recall, 10 each of CS + and CS- trials (CS + in the absence of US) are presented, interleaved. During reinstatement there are 2 trials each of CS + US and CS−, interleaved. The CS + is followed by the same US as conditioning. These trials are followed by a final re-extinction phase, which is identical to the extinction phase except that there are only 5 trials each of CS + and CS-. After each task phase, participants are asked to rate their nervousness in the same way as was done for part one of the paradigm. Inter-trial intervals and trace periods for the second part of the task are as per the first part of the task.

Note that the two faces are counterbalanced across participants as CS + or CS−. For all phases, CSs are pseudo-randomised such that there are no more than 2 consecutive trials of the CS + or CS−, and an equal number of CS + trials are presented in the first and last half of each block (same for CS-).

Following the scan (outside of the scanner), participants are presented with a one-question survey that assesses their awareness of the association between the CS + and the US.

#### Skin conductance response

Skin conductance response (SCR) is measured during the fear tasks of the MRI scan. SCR is collected by applying saline electrode gel to MRI-compatible electrodes that are placed on the participant’s index and middle fingers on their non-dominant hand with a Velcro strap, after being cleaned with an alcohol wipe. The response is then collected using an ADInstruments PowerLab 16/30 data acquisition unit paired with an ADInstruments galvanic skin response amplifier before SCR data is extracted using LabChart software (ADInstruments, version 8).

#### Saliva samples

 Adolescent participants are asked to provide saliva samples immediately after waking, before having anything to eat or drink, with help from their caregivers (all equipment provided). Female participants are asked to collect five saliva samples and male participants are asked to collect two; sample collections occur a week apart with the final day of collection to be the same day as participants’ in-person visit and MRI scan. If participants are not able to collect a sample on the dates we specify, they are asked to collect the saliva sample as soon as practical within a window of two days. Participants who are not able to collect a sample within the two-day window are asked to skip the sample that week, and resume collecting the sample the following week. Saliva samples are collected by passive drool using a straw into 10mL Techno Plas centrifuge tubes, before being stored in the participant’s freezer within a sealed Bio-Bottle^®^ until the day of their in-person visit. Participants are asked to record how long it takes the adolescent to reach the marked 2mL line on the tube and complete the Positive and Negative Affect Schedule [87] online to assess state mood at each sample collection. Participants also record any consumption of medication, caffeine, alcohol, or other drugs within the day prior to their sample. In sum, each sample (including questions) takes 5–10 min to collect. On the day of their in-person visit, families are asked to transport the Bio-Bottle containing the adolescent’s saliva samples inside a cooler bag packed with Techni-Ice (provided) and to minimise the time samples spend outside of the freezer. Saliva samples are checked by a researcher on arrival at the participants’ in-person visit, before being stored in a -30 °C freezer onsite until assay of hormones (i.e., E2 and P4).

At time of assay, samples will be defrosted and centrifuged, with the supernatant assayed for levels of E2 and P4. Salivary assays of these hormones are widely accepted valid substitutes for measuring non-protein bound serum levels [88]. Hormonal assays will be conducted by the Vaccine Immunology group at the MCRI, using Salimetrics Enzyme-Linked Immunosorbent Assay kits. Assays will be performed in duplicate, following manufacturer protocols.

#### Hair samples

Hair grows at a rate of approximately 1 cm per month [89], therefore a section of hair that is 3 cm in length provides an indication of hormonal output over several months. Hair samples are collected by a researcher at the in-person visit. The sample is taken from the posterior vertex of the scalp as it has the lowest coefficient of variation for hormonal levels compared with other areas of the scalp [90]. Researchers first isolate a section of scalp hair approximately 2cm^2^ by combing and clipping away excess hair, before looping a piece of string around the sample. Participants are shown a photo of the desired sample prior to collection, for confirmation of their approval. Samples are cut with hair-suitable scissors as close to the scalp as possible, with longer samples to be trimmed from the non-scalp end to fit within a folded foil pack (with the scalp end marked) without bending. Shorter hair samples (less than 3 cm in length) are stored untied. Samples are kept inside a resealable plastic bag and stored in an airtight container away from light onsite until assay. Hair samples will be processed and assayed using Salimetrics Enzyme-Linked Immunosorbent Assay kits for cortisol. In addition, researchers and participants also answer several questions about the hair, including the use of hair dye within the past 6 months, as well as frequency and recency of hair wash.

#### Anthropometry

Measurements of adolescent participants’ height, weight, and waist circumference are collected. Each measurement is taken twice (and the mean value utilised), unless the two values differ by ≥ 5 mm for height and waist circumference, or ≥ 0.1 kg for mass, in which case a third measurement would be obtained (and then the median value is utilised). Measurements are taken by the adolescent’s parent or caregiver with the instruction of the researcher present; however, upon request, measurements are taken by an experienced researcher. Participants are asked to remove shoes and any heavy items of clothing beforehand, and weight is measured to the nearest 0.1 kg using a Tanita HD-382 digital scale. Height is measured to the nearest 0.1 cm using a rigid stadiometer. Participants are informed that waist circumference can be measured over a light layer of clothing, if this would make them feel more comfortable. Waist circumference is measured to the nearest 0.1 cm using a medical tape measure.

### Anticipated data analysis

While we provide a general overview here, detailed methodologies and the specific analytical approaches will be published in the resulting empirical papers. The specific procedures for processing and analysing the data will be determined by the objectives and data subsets used in each individual empirical paper. We will endeavour to pre-register our analyses where possible and openly share scripts online, with details provided in the respective papers.

#### Power analysis

Given the large scope of our study, which includes a broad array of measures and encompasses several key aims and sub-aims, for the purposes of this protocol paper, we will highlight the power analysis related to our first aim. Power analyses for additional aims and sub-aims will be provided in subsequent empirical papers.

We illustrate power in an example analysis that is focused on estimating E2 variability in predicting PFC task-related function. Using Monte Carlo simulation (10000 draws) in Mplus 8, our anticipated sample of 145 female participants would provide 82% power to detect a true E2 variability effect of even small magnitude (i.e., β = 0.21, representing just ~ 4% unique extra variance explained in PFC function above a base level of 16% explained by any other variables in the model; at α = 0.05).

#### General statistical approaches

Linear regression, mixed effects models, and structural equation modelling will be used to address specific aims and hypotheses. For example, structural equation modelling will be used to examine the impact of parental factors on the brain and mental health for Aims 2.1 and 2.2, and random forest regression will be applied to analyse the impact of timing of adversity on the brain (Aim 2.1, Fig. 1). Analyses will include as covariates any variables deemed to confound associations of interest.

For null hypothesis significance testing, statistical thresholds will be set at *p* < .05 with corrections for multiple comparisons, though methods may vary with evolving standards. In brain imaging analyses, any region-based analyses will be supplemented with whole-brain or network-based analyses.

## Discussion

The PANDA study aims to address several key gaps in the field, including (1) improving knowledge regarding the role of sex hormones in the brain, specifically in regards to emotion processing, and how this is associated with anxiety during adolescence, and (2) understanding how these associations might be moderated by the social environment (particularly parental factors and previous exposure to ACEs).

Data gathered within PANDA will contribute to refining current models of the neurodevelopmental causes of mental health problems in young people, which have recently been suggested to be overly simplistic and speculative, with limited incorporation of the effects of puberty (and associated hormonal changes), in addition to being limited to focusing on depression and behavioural problems (at the expense of anxiety, which has higher prevalence). Parenting literature has primarily been an investigation of mothers only, and investigation of ACE exposure has rarely considered the timing of such events.

PANDA has several strengths. It will utilise repeated measurement of hormones (longitudinal saliva collection over a month) and will examine the developmental timing of ACEs, as well as both maternal and paternal parenting factors. It aims to address mechanistic questions through examination of brain function during key emotion processes implicated in the development of anxiety. It will also leverage multi-method, multi-informant collection of mental health symptoms. This study will enhance knowledge of the biological and environmental contributors to emotion dysregulation and anxiety in adolescents. Continued research is needed in this area to pave the way for future clinical research involving targeted hormonal or psychological treatments for at-risk female adolescents.

## Supplementary Information

Supplementary Material 1.

## Data Availability

No datasets were generated or analysed during the current study.

## Abbreviations

ACEs: Adverse Childhood Experiences
CS: Conditioned Stimuli
E2: Oestradiol
FER: Fear Extinction Recall
fMRI: Functional Magnetic Resonance Imaging
HPA: Hypothalamic–Pituitary–Adrenal
MINI-KID: Mini-International Neuropsychiatric Interview for Children and Adolescents
MRI: Magnetic Resonance Imaging
P4: Progesterone
PANDA: Puberty and NeuroDevelopment in Adolescents
PFC: Prefrontal Cortex
SCR: Skin Conductance Response
US: Unconditioned Stimuli
vmPFC: Ventromedial Prefrontal Cortex
