# Supplementary material for: Puberty and NeuroDevelopment in adolescents (PANDA): a study protocol
Source: BMC Pediatr. 2024 Nov 26;24:768. doi: 10.1186/s12887-024-05197-w (PMC11590350; doi:10.1186/s12887-024-05197-w)
Supplement: Supplementary file 1 — Supplementary Material 1. [file 12887_2024_5197_MOESM1_ESM.pdf]

Puberty and NeuroDevelopment in Adolescents (PANDA) Study Protocol Paper - Supplementary Materials

Measures

Demographics

Supplementary Table S1

Demographic Items

Item	Adolescent	Parent
Date of birth	✓ *	✓ *
Biological sex	✓ *	
Gender	✓ *	✓ *
Ethnicity and race	✓	
Country of birth	✓	✓ *
English first-language status	✓	
Relationship to child	NA	✓ *
Relationship to second parent	NA	✓
Living with child status	NA	✓ *
Total number of people in household		✓ *
Number of siblings (and birth order)	✓	
Birth older	✓	
Highest level of education		✓ *
Occupational status (and occupation if applicable)		✓ *
Family/household income (including changes since COVID-19 pandemic)		✓ *

Note. Column headings indicate if information is gathered about the adolescent or about their parent. The same information is also asked to the second parent if they are involved in the study.
* Item included within the online study.

Questionnaire Measures

Anxiety and mood-related questionnaires

Beck Anxiety Inventory (BAI; 1)

The BAI is a widely used self-report instrument for detecting anxiety symptoms in adults. It is a brief, 21- item, criteria-referenced assessment for measuring the intensity of anxiety in clinical and non-clinical participants over the prior month. The measure is demonstrated to have good validity, and high internal consistency and test re-test reliability.

Brief Multidimensional Students' Life Satisfaction Scale (BMSLSS; 2)

The BMSLSS measures life satisfaction in children and adolescents. This brief form of the instrument consists of only 6 items and asks children to rate items on a six-point scale from 'strongly disagree' to 'strongly agree'. This instrument has good validity and internal consistency.

Center for Epidemiologic Studies Depression Scale (CESD; 3)

The CESD is a screening test for depression and depressive disorder. The CESD measures symptoms defined by the American Psychiatric Association' Diagnostic and Statistical Manual (DSM-V) for a major depressive episode. The CESD is a brief questionnaire, which consists of 20 items and checks for anhedonia, dysphoria, fatigue among other things.

Child Depression Inventory – 2 (CDI-2; 4)

The CDI assesses depressive symptoms in children and adolescents, using both self-report and parent report. This 28-item measure has both a self-report form (CDI:2- SR) and a parent-report form (CDI2:P) with corresponding items that are suitably rephrased. The parent report form is focused on observable manifestations of depression to maximize validity. This instrument is widely used and has good validity and internal consistency.

Childhood Anxiety Sensitivity Index (CASI; 5)

The CASI is an 18-item version of the Anxiety Sensitivity Index, specially adapted for use in children. This instrument measures the extent to which the adolescent believes experiencing anxiety will result in negative consequences, using items such as the following: "It scares me when my heart beats fast", and "I don't want other people to know when I feel afraid". Children respond to the items on a 3-point scale: 1(*none*), 2 (*some*), or 3 (*a lot*). Total scores are calculated for the measure, ranging from 18-54, with higher scores indicating higher levels of anxiety sensitivity. This measure has good internal consistency and test-retest reliability for both clinical and nonclinical samples.

Positive and Negative Affect Schedule (PANAS; 6,7)

PANAS is a self-report measure of affect. It contains 20 items, 10 for positive affect and 10 for negative affect. Each item is rated from 1 (very slightly or not at all) to 5 (extremely). Positive affect includes items to measure the extent to which a person feels interested, excited, strong, enthusiastic, proud, alert, inspired, determined, attentive, and active while negative affect reflects how stressed, upset, guilty, scared, hostile, irritable, ashamed, nervous, jittery, and afraid a person feels. In the current study, participants are asked to complete the PANAS concurrent with each saliva collection in addition to one week following the MRI scan. Participants are asked to rate their affect over the past week. The PANAS is demonstrated to be a valid measure with good internal consistency and test-retest reliability.

Spence Children's Anxiety Scale-Short (SCAS-S; 8)

The SCAS-S is a questionnaire designed to measure the severity of anxiety symptoms in children, following the dimensions of a DSM-IV anxiety disorder. It is specially designed to be easy and quick for children to complete. This measure consists of 19 items in total, covering generalized anxiety disorder, separation anxiety disorder, social anxiety disorder, specific phobias and panic disorder. The SCAS-S is a widely used in clinical and research contexts, and has good internal reliability, test-retest reliability, and construct validity.

State-Trait Anxiety Inventory (STAI; 9)

The STAI is a commonly used measure of state and trait anxiety in adults, using two 20-item scales. Individuals must answer items on a 4-point Likert scale, where higher scores indicate higher anxiety. The State scale assesses state anxiety, which is shorter-term and often more situation-dependent, while the Trait scale measures trait anxiety, which his more long-term and relates to how an individual generally feels. These two scales yield individual scores, to determine which type of anxiety may be more dominant in the individual. This measure demonstrates good internal consistency and test-retest reliability.

State-Trait Anxiety Inventory for Children (STAIC; 10)

The STAIC measures state and trait anxiety using two 20-item scales. Children must answer whether statements apply on a scale from 'hardly ever true' to 'often true'. The State scale assesses state anxiety, which is shorter-term and often more situation-dependent, while the Trait scale measures trait anxiety, which is more long-term and relates to how a child generally feels. These two scales yield individual scores to determine which type of anxiety may be more dominant in the child. This measure demonstrates good internal consistency and test-retest reliability.

The Strengths and Difficulties Questionnaire (SDQ; 11)

The SDQ is a brief behavioural screening questionnaire for children aged 2-17 years old. It contains 5 subscales (Emotional Symptoms; Conduct Problems; Hyperactivity/Inattention; Peer Relationship Problems; Prosocial Behaviour), each with 5 items. Externalising behaviours can be captured by summing Hyperactivity and Conduct Problem subscales, which was implemented for the PANDA study. Similar to that of internalising problems, there is evidence that parental psychopathology and/or parenting behaviours may influence the onset of externalising problems in youth, and that there may be neurobiological mechanisms which underpin this relationship. Therefore, externalising behaviours are also captured.

Visual Analog Scale (VAS; 12) for assessment of anxiety surrounding MRI assessment

The VAS provides an easy way of visually assessing how a participant is feeling throughout their in-person appointment. Adolescents are asked by a researcher to mark a horizontal line ranging from "not at all" to "a lot", to indicate how they feel right now across four emotions: nervous,

upset, interested, and happy. This measure is used to assess how children feel before and after their mock scan, and after their MRI scan.

Puberty

Pubertal Development Scale (PDS; 13)

The PDS is a widely used instrument for assessing pubertal development of children and adolescents. The self-report version of the PDS is used in the current study, in which individuals report on physical characteristics of puberty, such as body hair growth and breast development. The PDS consist of five items each for males and females, in which physical development is rated from 1 (not yet started) to 4 (complete). There is an additional item for females assessing menarche.

Where it is detected that an adolescent's biological sex and gender are not congruent, the adolescent is presented with a question to determine their comfort in answering questions regarding their biological sex, in reference to pubertal development. Adolescents are instructed that if they chose ‘Yes’ and proceed to the PDS module, they can change their mind and skip questions. Those that choose ‘No’ are not presented with the PDS module.

Attachment and parenting

Children’s Perceptions of Interparental Conflict (CPIC; 14): Child Report

The CPIC aims to assess the perspective of children and adolescents regarding the conflict between their parents/caregivers. Respondents are asked to rate items on a 3-point Likert scale (1 = “True”, 2 = “Sort of true”, 3 = “False); items include prompts such as ‘They may not think I know it, but they argue or disagree a lot’. This study includes two subscales of interparental conflict, frequency and intensity (13 items), enabling us to gain further insight into the family environment. This allows for the establishment of the relationship with the parents/caregivers of the child, and whether the home environment may be one which further influences the child’s internalising. The CPIC has demonstrated good internal consistency and test-retest reliability.

Egna Minnen Betraffande Uppfostran (EMBU-C; 15–18): Child Report/Parent Report.

The EMBU addresses multiple factors of parenting, including rejection, emotional warmth and overprotection. The current study administers the ‘Anxious Rearing’ sub-scale (10 items), which is captured through the broader theme of overprotection. Literature suggests that modelling of anxious behaviour is important for fear/threat learning. Respondents are asked to rate items on a 4-point Likert scale (1= “No, never”, 2 = “Yes, but seldom”, 3 = “Yes, often”, 4 = “Yes, most of the time”); items include prompts such as ‘My parent/caregiver... worries about you doing dangerous things’. The EMBU has shown good internal consistency and test re-test reliability.

Experience in Close Relationships - Revised (ECR; 19)

The ECR-R is a widely used self-report measure of romantic attachment in adult relationships. Individuals must indicate on a 7-point Likert scale, from “strongly disagree” to “strongly agree”, how much each statement applies to how generally experience romantic relationships, not one particular relationship. The current study administers the 18-item Anxiety subscale of the ECR-R. This measure demonstrates good validity and internal consistency.

Experience in Close Relationships- Revised for Children (ECR-RC) – short version (20)

The ECR-RC is a commonly used self-report instrument for assessing child and adolescent attachment anxiety (21). More recently, an abridged version has been developed with 12 items instead of 36 (20). It has excellent reliability and validity. Individuals must indicate on a 7-point Likert scale ranging from “strongly disagree” to “strongly agree” how much each statement applies to how they feel regarding the relationship with their parent. This instrument presents the same items twice- once for the relationship with the mother (or first parent/caregiver), once for the relationship with the father (or second parent/caregiver).

Inclusion of Other in Self (IOS; 22)

The IOS is a single-item measure of how close the respondent feels with another person or group. This visual measure presents seven pairs of circles, ranging from just touching and overlapping increasingly more until they are almost completely overlapping. One circle is labelled ‘self’, and the second circle ‘other’, representing the respondent and other person. We use this instrument as a measure of closeness between the child/adolescent and each of their parents, as well as for each parent between themselves and the other parent (as romantic partners).

Parental Acceptance-Rejection Questionnaire (PARQ; 23): Parent and Child Report

The PARQ is a 24-item measure validated for use in parents, children and infants. The 5 factors (Undifferentiated Rejection; Indifference/Neglect; Hostility/Aggression; Warmth/Affection; Control) within this measure are suggested to influence child internalising. The PARQ was designed to address the limitations of the Child’s Report of Parent Behaviour Inventory (CRPBI) and includes more factors to investigate parent child relationships. We have chosen to omit the ‘control’ sub-scale, as it is believed this factor is better suited to predicting and investigating externalising symptoms. Respondents are asked to rate items on 4-point Likert scale (1 = “Almost always true of me”, 2 = “Sometimes true of me”, 3 = “Rarely true of me”, 4 = “Almost never true of me”) where items for parents/caregivers include prompts such as, ‘I care about what my child thinks and encourage her/him to talk about it’. Prompts for children include items such as, ‘My parent/caregiver makes it easy for me to tell them things that are important to me’.

Adverse experiences

Maltreatment and Abuse Chronology of Exposure (MACE; 24,25) – Adolescent-Report

The MACE is a 52-item self-report questionnaire that assesses exposure to ten types of maltreatment (i.e., emotional neglect, non-verbal emotional abuse, parental physical maltreatment, parental verbal abuse, peer emotional abuse, peer physical bullying, physical neglect, sexual abuse, witnessing interparental violence, and witnessing violence to siblings) during childhood. For each experience endorsed, respondents are asked to indicate the ages at which they were exposed to the experience. The MACE has undergone extensive psychometric testing and has been validated in an adult sample (24). The authors reported good to excellent psychometric properties (including test-retest reliability; (24). We have slightly altered some wording to be more appropriate for adolescents. Note that the MACE has been previously used in adolescent populations (25).

Maltreatment and Abuse Chronology of Exposure (MACE) – Parent-Report

The MACE (parent-report) is an adapted version of the scale described above, designed to capture a child's exposure to ten types of maltreatment, as reported by a parent. It contains 52 items, which ask parents to indicate if their child (who is participating in the ELM study) has been exposed to certain adverse experiences, and to indicate the ages of exposure for each endorsed experience. We use this parent-report-version of the MACE to obtain comparable data, and because self-report of very early experiences could be subject to infantile amnesia.

Social and emotional functioning

Adolescent Measure of Empathy and Sympathy (AMES; 26)

A self-report measure of cognitive empathy, affective empathy, and sympathy. Each subscale contains 4 items, for a total of 12 items, rated on a 5-point Likert scale. An example of an item measuring affective sharing is "When a friend is angry, I feel angry too". The other feelings addressed by the affective sharing subscale are sadness, fear, and nervousness. An example of an item measuring cognitive empathy is "I can easily tell how others are feeling". An example of an item measuring empathic concern is "I feel sorry for someone who is treated unfairly". This measure has been validated in 10- to 15-year-olds, and has robust psychometrics, including satisfactory internal consistency, and test-retest reliability over two weeks (26). The cognitive empathy and sympathy subscales were validated against the perspective taking and empathic concern subscales of a well-used adult empathy self-report measure (the Interpersonal Reactivity Index).

Difficulties in Emotion Regulation Scale – Short Form (DERS-SF; 27)

The DERS (28) measures emotion regulation problems. It is well-validated and widely used in adults and adolescents. A short form of this scale was created by Kaufman et al. (27) containing 18 items and the same six subscales as the long form; difficulties engaging in goal-directed behaviour, impulse control difficulties, lack of emotional awareness, limited access to emotion regulations strategies, lack of emotional clarity. Items, for example, "When I'm upset, I have

difficulty concentrating” are rated from 1 = *Almost never* to 5 = *Almost always.* The DERS-SF has been validated in adolescents (ages 12-20) and has sound psychometric properties; including high correlation to the full-length version (subscales: .91 to .98), and good internal consistency (α for subscales: between .79 and .91).

Empathetic Distress Questionnaire (29,30)

This questionnaire is a revised version of the Empathetic Distress Questionnaire (29). It has been used in adolescents aged 12-18 (30). The measure includes 18 items and measures the extent to which one shares in the distress of another (for example a friend), and takes on the distress of another, as if it were their own. Example items include “I felt as though my friend's upset feelings became my upset feelings too” and “I felt as upset about my friend's problem as if it were my own.” Participants indicated how well each item described them using a 5-point Likert scale ranging from does not describe me at all (0) to describes me very well (4). The internal reliability was excellent for the revised empathetic distress measure (Cronbach's α = .96) (30).

Additional measures

Edinburgh Handedness Inventory (EDI) short form (31)

The short-form version of the EDI is a 4-item measure of handedness. This instrument asks which hand (left or right) the respondent typically uses when performing typical daily tasks.

Additional Information Regarding Procedures

Recruitment strategy

In February 2023, Meta introduced new restrictions for advertising that targeted adolescents under the age of 18 across its platforms (i.e., Instagram, Facebook). In sum, these restrictions limited targeting to broad location and age whereas previously, advertisers could also include interest, gender, and greater location specificity (i.e., in Melbourne, Australia). Considering these incoming restrictions and to implement our recruitment strategy most effectively, we removed any advertisement targets that were set to adolescents from February 2023. It should be noted that adolescent advertising through social media was aimed at recruiting the upper end of the age demographic, and any social media recruitment for younger adolescents (especially those under 13 years old, per Meta policy) has always included parents. This strategy aims to reduce selection bias by ensuring broad accessibility and reaching a diverse participant pool.

Reimbursement

Participants attending in person appointments are reimbursed at a rate of $20AUD per hour for their participation, which is also extended to the parent or caregiver who attends the appointment with the adolescent participant. Additionally, so as not to create a financial barrier to participation for families (mitigating selection bias), parking vouchers for the Royal Children’s Hospital are provided to those who park on site, and travel reimbursement for alternative means of transport can be supplied on request if required.

Risk management

Researchers working with adolescents have undergone relevant training (i.e., Applied Suicide Intervention Skills Training; ASIST) and partake in ongoing supervision with a clinical psychologist to identify and appropriately handle risk situations that arise. When indications of potentially clinically significant psychopathology or risk to the participant's safety are present in questionnaires or the online interview, researchers follow up with a comprehensive risk assessment and debrief with adolescents and their parents, as appropriate.

MRI

Mock MRI procedure

Adolescent participants practice the MRI procedure with a mock scan replicating the MRI to help them feel more comfortable. Researchers provide safety information and explain the relevant parts to the scan while participants lie down on the gantry and practice going inside the scanner. During the mock scan, researchers assess the participant’s capacity to complete the real scan, taking observed anxiety and movement levels into consideration. Researchers ask participants to complete a Visual Analogue Scale (VAS; 12) prior to and following the mock, and after their MRI scan. In addition, prior to the scan, parents are required to complete an MRI safety form which is subsequently reviewed by the MRI radiography team to ensure the safety of adolescent participants.

Fear learning fMRI task

Initially, it was planned for all age groups to complete the fear learning fMRI task (previous studies (32,33) successfully used this task in this age group). However, the research team made the decision to no longer run the fMRI task for the younger half of the cohort. This decision followed the opening of recruitment to include younger adolescents between the ages of 11-13 (after recruitment had already commenced with 14-16-year-olds) where researchers observed an increase in distress surrounding this specific task. This was evidenced by verbal feedback from adolescents and parents, and 23% of the first 13 adolescents to undertake the fMRI fear learning task within the younger half of the cohort reporting distress or asking to stop the task entirely. In prioritising the beneficence of participation, the decision was made to no longer run the task for the younger participants. It should be noted that for any adolescents who find the MRI distressing in any way, researchers provide a comprehensive debrief with adolescents and their parents, as appropriate.

Online Study Assent Comprehension Check Questions

This check in required adolescents to correctly respond to the following three questions, before they could proceed:

1. Is my participation in this study voluntary?

   a. No, I must complete the study,
   b. Yes, it is perfectly ok if I don’t agree to be in this study, and I can stop taking part at any time,

c. Yes, however once I start the study, I have to complete it

2. Which of the following is correct?

a. There is a chance that I could feel a bit upset after answering the survey questions. My answers are confidential, unless I provide information that indicates that I or someone else may be at risk of harm,
b. There is a chance that I could feel a bit upset after answering the survey questions, and my answers are not confidential,
c. There is no chance that I could feel upset after answering the survey questions. My answers are confidential, unless I provide information that indicates that I or someone else may be at risk of harm.

3. Who will be participating in the study?

a. The study only requires me to complete a questionnaire,
b. The study requires that one parent and I complete questionnaires,
c. The study requires that both of my parents and I complete questionnaires.

Similar checkpoints have been used in online-only studies which target youth, to ensure that they grasp what they are assenting to. Failure to correctly answer these questions resulted in suspension of their involvement, and exit of the questionnaire, to prevent uninformed consent and participation.

STROBE Statement—Checklist of items that should be included in reports of *cross-sectional studies*

NB. Some items have been noted as "NA", for not applicable, as they relate to cross-sectional study empirical papers, rather than the current protocol paper.

	**Item No**	**Recommendation**	**Completed**
**Title and abstract**	1	(*a*) Indicate the study's design with a commonly used term in the title or the abstract	✓
		(*b*) Provide in the abstract an informative and balanced summary of what was done and what was found	✓
**Introduction**			
Background/rationale	2	Explain the scientific background and rationale for the investigation being reported	✓
Objectives	3	State specific objectives, including any prespecified hypotheses	✓

**Methods**			
Study design	4	Present key elements of study design early in the paper	✓
Setting	5	Describe the setting, locations, and relevant dates, including periods of recruitment, exposure, follow-up, and data collection	✓
Participants	6	(*a*) Give the eligibility criteria, and the sources and methods of selection of participants	✓
Variables	7	Clearly define all outcomes, exposures, predictors, potential confounders, and effect modifiers. Give diagnostic criteria, if applicable	✓
Data sources/ measurement	8*	For each variable of interest, give sources of data and details of methods of assessment (measurement). Describe comparability of assessment methods if there is more than one group	✓
Bias	9	Describe any efforts to address potential sources of bias	✓
Study size	10	Explain how the study size was arrived at	✓
Quantitative variables	11	Explain how quantitative variables were handled in the analyses. If applicable, describe which groupings were chosen and why	✓
Statistical methods	12	(*a*) Describe all statistical methods, including those used to control for confounding	✓
		(*b*) Describe any methods used to examine subgroups and interactions	NA
		(*c*) Explain how missing data were addressed	NA
		(*d*) If applicable, describe analytical methods taking account of sampling strategy	NA
		(*e*) Describe any sensitivity analyses	NA
**Results**			

Participants	13	(a) Report numbers of individuals at each stage of study—eg numbers potentially eligible, examined for eligibility, confirmed eligible, included in the study, completing follow-up, and analysed	NA
		(b) Give reasons for non-participation at each stage	NA
		(c) Consider use of a flow diagram	NA
Descriptive data	14	(a) Give characteristics of study participants (eg demographic, clinical, social) and information on exposures and potential confounders	NA
		(b) Indicate number of participants with missing data for each variable of interest	NA
Outcome data	15	Report numbers of outcome events or summary measures	NA
Main results	16	(*a*) Give unadjusted estimates and, if applicable, confounder-adjusted estimates and their precision (eg, 95% confidence interval). Make clear which confounders were adjusted for and why they were included	NA
		(*b*) Report category boundaries when continuous variables were categorized	NA
		(*c*) If relevant, consider translating estimates of relative risk into absolute risk for a meaningful time period	NA
Other analyses	17	Report other analyses done—eg analyses of subgroups and interactions, and sensitivity analyses	NA
**Discussion**			
Key results	18	Summarise key results with reference to study objectives	NA
Limitations	19	Discuss limitations of the study, taking into account sources of potential bias or imprecision. Discuss both direction and magnitude of any potential bias	NA
Interpretation	20	Give a cautious overall interpretation of results considering objectives, limitations, multiplicity of analyses, results from similar studies, and other relevant evidence	NA
Generalisability	21	Discuss the generalisability (external validity) of the study results	NA
**Other information**			

Funding	22	Give the source of funding and the role of the funders for the present study and, if applicable, for the original study on which the present article is based	✓

Note: An Explanation and Elaboration article discusses each checklist item and gives methodological background and published examples of transparent reporting. The STROBE checklist is best used in conjunction with this article (freely available on the Web sites of PLoS Medicine at http://www.plosmedicine.org/, Annals of Internal Medicine at http://www.annals.org/, and Epidemiology at http://www.epidem.com/). Information on the STROBE Initiative is available at www.strobe-statement.org.

References:

1. Beck AT, Epstein N, Brown G, Steer RA. An inventory for measuring clinical anxiety: Psychometric properties. J Consult Clin Psychol. 1988;56(6):893–7.

2. Seligson JL, Huebner ES, Valois RF. Preliminary Validation of the Brief Multidimensional Students' Life Satisfaction Scale (BMSLSS). Soc Indic Res. 2003 Feb 1;61(2):121–45.

3. Radloff LS. The CES-D Scale: A Self-Report Depression Scale for Research in the General Population. Appl Psychol Meas. 1977 Jun 1;1(3):385–401.

4. Kovacs M. Children's Depression Inventory (CDI and CDI 2). In: The Encyclopedia of Clinical Psychology [Internet]. John Wiley & Sons, Ltd; 2015 [cited 2024 Jul 16]. p. 1–5. Available from: https://onlinelibrary.wiley.com/doi/abs/10.1002/9781118625392.wbecp419

5. Silverman WK, Fleisig W, Rabian B, Peterson RA. Childhood Anxiety Sensitivity Index. J Clin Child Psychol. 1991 Jun 1;20(2):162–8.

6. Crawford JR, Henry JD. The Positive and Negative Affect Schedule (PANAS): Construct validity, measurement properties and normative data in a large non-clinical sample. Br J Clin Psychol. 2004;43(3):245–65.

7. Watson D, Clark LA, Tellegen A. Development and validation of brief measures of positive and negative affect: The PANAS scales. J Pers Soc Psychol. 1988;54(6):1063–70.

8. Ahlen J, Vigerland S, Ghaderi A. Development of the Spence Children's Anxiety Scale - Short Version (SCAS-S). J Psychopathol Behav Assess. 2018 Jun 1;40(2):288–304.

9. Spielberger CD. Manual for the State-Trait Anxiety Inventory (STAI). Palo Alto CA Consult Psychol Press. 1983;

10. Spielberger CD, Edwards CD, Montouri J, Lushene R. State-Trait Anxiety Inventory for Children [Internet]. 1973 [cited 2024 Jul 3]. Available from: http://doi.apa.org/getdoi.cfm?doi=10.1037/t06497-000

11. Goodman R. Strengths and Difficulties Questionnaire (SDQ) [Database record]. APA PsycTests. [Internet]. 1997 [cited 2024 Jul 3]. Available from: https://doi.apa.org/doi/10.1037/t00540-000

12. Lesage FX, Berjot S, Deschamps F. Clinical stress assessment using a visual analogue scale. Occup Med. 2012 Dec 1;62(8):600–5.

13. Petersen AC, Crockett L, Richards M, Boxer A. Pubertal Development Scale (PDS) [Internet]. 2014 [cited 2024 Jul 8]. Available from: https://doi.apa.org/doi/10.1037/t06349-000

14. Grych JH, Seid M, Fincham FD. Assessing Marital Conflict from the Child's Perspective: The Children's Perception of Interparental Conflict Scale. Child Dev. 1992;63(3):558–72.

15. Muris P, Meesters C, van Brakel A. Assessment of Anxious Rearing Behaviors with a Modified Version of “Egna Minnen Beträffande Uppfostran” Questionnaire for Children. J Psychopathol Behav Assess. 2003 Dec 1;25(4):229–37.

16. Wei C, Kendall PC. Parental Involvement: Contribution to Childhood Anxiety and Its Treatment. Clin Child Fam Psychol Rev. 2014 Dec 1;17(4):319–39.

17. Young BJ, Wallace DP, Imig M, Borgerding L, Brown-Jacobsen AM, Whiteside SPH. Parenting Behaviors and Childhood Anxiety: A Psychometric Investigation of the EMBU-C. J Child Fam Stud. 2013 Nov 1;22(8):1138–46.

18. Affrunti NW, Woodruff-Borden J. The Roles of Anxious Rearing, Negative Affect, and Effortful Control in a Model of Risk for Child Perfectionism. J Child Fam Stud. 2017 Sep 1;26(9):2547–55.

19. Fraley RC, Waller NG, Brennan KA. An item response theory analysis of self-report measures of adult attachment. J Pers Soc Psychol. 2000;78(2):350–65.

20. Brenning K, Van Petegem S, Vanhalst J, Soenens B. The psychometric qualities of a short version of the Experiences in Close Relationships Scale – Revised Child version. Personal Individ Differ. 2014 Oct 1;68:118–23.

21. Brenning K, Soenens B, Braet C, Bosmans G. An adaptation of the experiences in close relationships scale-revised for use with children and adolescents. J Soc Pers Relatsh. 2011;28(8):1048–72.

22. Aron A, Aron EN, Tudor M, Nelson G. Close relationships as including other in the self. J Pers Soc Psychol. 1991;60(2):241–53.

23. Rohner RP, Khaleque A. Parental acceptance-rejection questionnaire (PARQ): Test manual. Handb Study Parent Accept Rejection. 1997;4:43–106.

24. Teicher MH, Parigger A. The ‘Maltreatment and Abuse Chronology of Exposure’ (MACE) Scale for the Retrospective Assessment of Abuse and Neglect During Development. PLOS ONE. 2015 Feb 25;10(2):e0117423.

25. Kim-Spoon J, Herd T, Brieant A, Peviani K, Deater-Deckard K, Lauharatanahirun N, et al. Maltreatment and brain development: The effects of abuse and neglect on longitudinal trajectories of neural activation during risk processing and cognitive control. Dev Cogn Neurosci. 2021 Apr 1;48:100939.

26. Vossen HGM, Piotrowski JT, Valkenburg PM. Development of the Adolescent Measure of Empathy and Sympathy (AMES). Personal Individ Differ. 2015 Feb 1;74:66–71.

27. Kaufman EA, Xia M, Fosco G, Yaptangco M, Skidmore CR, Crowell SE. The Difficulties in Emotion Regulation Scale Short Form (DERS-SF): Validation and Replication in Adolescent and Adult Samples. J Psychopathol Behav Assess. 2016 Sep 1;38(3):443–55.

28. Gratz KL, Roemer L. Multidimensional Assessment of Emotion Regulation and Dysregulation: Development, Factor Structure, and Initial Validation of the Difficulties in Emotion Regulation Scale. J Psychopathol Behav Assess. 2004 Mar 1;26(1):41–54.

29. Smith RL, Rose AJ. The “cost of caring” in youths’ friendships: Considering associations among social perspective taking, co-rumination, and empathetic distress. Dev Psychol. 2011;47(6):1792–803.

30. Smith RL. Adolescents’ emotional engagement in friends’ problems and joys: Associations of empathetic distress and empathetic joy with friendship quality, depression, and anxiety. J Adolesc. 2015 Dec 1;45:103–11.

31. Oldfield RC. The assessment and analysis of handedness: The Edinburgh inventory. Neuropsychologia. 1971 Mar 1;9(1):97–113.

32. Lau JY, Britton JC, Nelson EE, Angold A, Ernst M, Goldwin M, et al. Distinct neural signatures of threat learning in adolescents and adults. Proc Natl Acad Sci. 2011;108(11):4500–5.

33. Reinhard J, Slyschak A, Schiele MA, Andreatta M, Kneer K, Reif A, et al. Fear conditioning and stimulus generalization in association with age in children and adolescents. Eur Child Adolesc Psychiatry. 2022 Oct 1;31(10):1581–90.
